# Depression, anxiety and stress symptoms in patients presenting with dyspepsia at a regional hospital in KwaZulu-Natal province

**DOI:** 10.4102/sajpsychiatry.v25i0.1382

**Published:** 2019-10-21

**Authors:** Sijabulisiwe J. Tshabalala, Andrew Tomita, Suvira Ramlall

**Affiliations:** 1 Department of Psychiatry, Nelson R. Mandela School of Medicine, College of Health Sciences, University of KwaZulu-Natal, Durban, South Africa; 2 Centre for Rural Health, School of Nursing and Public Health, University of KwaZulu-Natal, Durban, South Africa; 3 KwaZulu-Natal Research Innovation and Sequencing Platform, Nelson R. Mandela School of Medicine, College of Health Sciences, University of KwaZulu-Natal, Durban, South Africa

**Keywords:** dyspepsia, depression, anxiety, stress, gut-brain

## Abstract

**Background:**

Depression, anxiety and stress (DAS) have been shown to be co-morbid with dyspepsia. Local data on the factors associated with these co-morbidities could inform the role of psychiatric intervention in affected patients.

**Aim:**

The aim of this study was to describe the frequency of undiagnosed DAS and their associated protective and risk correlates in a sample of patients undergoing endoscopies for dyspepsia.

**Setting:**

The study was conducted at a regional hospital’s gastro-intestinal unit in KwaZulu-Natal province.

**Method:**

A cross-sectional survey was conducted on 201 in- and outpatients with symptoms of dyspepsia awaiting endoscopy. Information on DAS symptomatology (using the DASS-21 screening questionnaire, as well as socio-demographic and clinical data) were collected.

**Analyses:**

Following a descriptive analysis of the participants’ socio-demographic and clinical details, linear regression models were fitted to identify potential risk and protective correlates linked to DAS symptomatology.

**Results:**

The mean age of participants (*N* = 201) was 48.89 years, of whom approximately two-thirds (*n* = 133; 66.17%) were women, 97% (*n* = 195) were African and 64.68% (*n* = 130) resided in rural areas. Anxiety was the most prevalent symptom category (*n* = 149; 74.13%) versus depression (*n* = 96; 47.76%) and stress (*n* = 68; 33.83%) in each category of symptom (mild to extremely) severity. In the severe and extremely severe range, anxiety existed without co-morbid depression or stress in 61.19% of anxious patients. Alcohol use was significantly associated with all three symptom categories (*p* < 0.01).

**Conclusions:**

Given high frequencies of depression and anxiety in patients undergoing endoscopies for dyspepsia, screening for common mental disorders is essential.

## Introduction

The high prevalence of psychological distress among hospital outpatients in South Africa necessitates an understanding of the comorbid depressive and anxiety disorders in those with chronic medical illness^1^ if their associated clinical and financial burdens are to be addressed. Dyspepsia is one chronic medical illness that is often overlooked in developing country settings.

Dyspepsia is a common presenting symptom complex in primary care all over the world,^2^ accounting for 5% of visits to general practitioners in the United States.^3^ Dyspepsia refers to a group of upper gastrointestinal symptoms with functional dyspepsia (FD) that are characterised by chronic pain and burning and early satiety, and for which no organic basis has been established.^4^ There have been changes to the internationally accepted definition of dyspepsia over the last 20 years, including to the Rome diagnostic criteria,^5^ which has implications for the estimated global prevalence of uninvestigated dyspepsia of 20.8%, and 5% – 11% for functional dyspepsia.^6^ While a revision of the diagnostic criteria in the 2016 Rome IV diagnostic criteria will have implications for future FD prevalence rates,^7^ figures as high as 60% have been reported from Jordan,^8^ 21% from the United Kingdom^9^ and 26% from the United States.^10^ According to Ghoshal et al.,^11^ 8% – 23% of Asian people suffer from FD and a rate of 45% has been reported in one sample of a Nigerian community.^12^ A range of organic pathologies may be found in 20% – 30% of those with dyspepsia, the most common of which is peptic ulcers (20%).^5^ The assumption is that dyspepsia in developing countries is mostly organic in nature compared to the more prevalent functional dyspepsia in developed countries.^13^

Risk factors associated with dyspepsia include *Helicobacter pylori* infection^14^, psychiatric disorders^15^ and behavioural factors^16^, while functional dyspepsia has been associated with anxiety, which sometimes precedes its onset. In a 10-year prospective study, anxiety at baseline, but not depression, increased the risk for developing FD by a factor of 7.6.^17^ In a study of a small sample of patients with dyspepsia, 82.5% were found to have anxiety, 60% depression and 67.5% had stress as measured by the Depression, Anxiety and Stress scale (DASS), while inflammatory changes on endoscopy were more frequently found in those with prominent anxiety symptoms.^18^ In a community survey of more than 2000 respondents, 8.0% met the Rome III diagnostic criteria for dyspepsia and had a two- and threefold increased risk of DSM-IV-TR (Diagnostic and Statistical Manual of Mental Disorders: Text Revision) Generalised Anxiety Disorder (GAD) and Major Depressive Episode, respectively. The psychiatric diagnoses often coincided with the onset of dyspepsia, with co-morbidity predicting the need for frequent medical consultations and high costs related to investigations.^19^

On psychometric tests, FD patients scored higher on anxiety, depression and somatisation than those without abdominal symptoms, suggesting the pathological central processing of visceral stimuli.^20^ Common mental disorders (CMDs) were found to be significantly more frequent in FD patients (71.33%), specifically females, than in controls (15.33%; *p* < 0.001).^21^

A bidirectional gut-brain network that links emotional, cognitive and gut functions is suggested by the high co-morbidity between gastrointestinal (GI) and psychiatric disorders and the frequent use of antidepressants to treat GI conditions.^22^ Antidepressants have been shown to be beneficial for some patients with dyspepsia.^23^ It has been postulated that a depressed mood is linked to altered immune-endocrine intestinal homeostasis and may be linked to gut microbiome.^24^ A prospective study of 1900 respondents found that higher baseline anxiety and depression predicted FD, with a bidirectional pathway being supported in one-third of individuals where a mood disorder preceded functional gastrointestinal disorders (FGID), and in two-thirds where the GI disorder preceded the mood disorder.^25^

Dyspepsia carries considerable direct and indirect (absenteeism and presenteeism) costs.^26^ Total mean direct annual cost per patient has been estimated at US $699, which includes over-the-counter medications, medical-aid copayments and alternative therapies. A mean of 1.4 h absence from work a week was also reported in a study that extrapolated the total population costs of functional dyspepsia in the United States in 2009 as being $18.4 billion.^27^ Based on payroll and medical health insurance data of more than 300 000 employees over a 4-year period, those with FD had greater medical costs and absenteeism and lower productivity than the controls.^28^ Despite its importance, dyspepsia has been not been the subject of much investigation in developing countries, especially in relation to psychiatric co-morbidity. The aim of this study was therefore to describe the frequency of depression, anxiety and stress (DAS) and their associated preventive and risk correlates in patients undergoing endoscopy for dyspepsia.

## Methods

A quantitative descriptive cross-sectional survey was conducted at a public sector, regional hospital’s GI unit in KwaZulu-Natal (KZN) province, South Africa. Ethical approval was obtained from the Biomedical Research Ethics Committee of the University of KwaZulu-Natal (BE 374/16). Permission to conduct the research was obtained from the KwaZulu-Natal Department of Health and the hospital management.

### Participants

The study population consisted of all in- and outpatients undergoing endoscopy who were referred from the hospital’s medical and surgical departments. For the purposes of this study, dyspepsia was defined as at least 3 months of one or more of the following symptoms: bothersome postprandial fullness, early satiation, epigastric pain and burning. In those with functional dyspepsia, there was no evidence of structural disease (including upper endoscopy) that is likely to explain the symptoms. Patients less than 40 years old, who had no improvement after 6 weeks of antacid treatment and first-line eradication therapy, were booked for endoscopy.

If the patient was more than 40 years old, the endoscopy was booked on the day of consultation to exclude possible organic causes and malignancy. Tests for bacteria were not conducted in patients with dyspepsia prior to the endoscopy due to the high prevalence of *H. pylori* and the financial constraints in our public health sector. The inclusion criteria were the following: patients with dyspepsia who had an endoscopy, aged 18 years and older and able to give consent, while those with severe medical or physical ailments, or currently taking medication for depression and/or anxiety, were excluded.

### Data collection and instruments

Data were collected between April and June 2017, with informed consent being obtained from all participants meeting the study inclusion criteria based on convenience sampling. All participants completed a customised demographic data sheet and the DASS-21 questionnaire to measure DAS symptomatology^29^ with the assistance of a field worker, and their clinical data were accessed from the hospital records.

The DASS-21^29^ is a 21-item self-reporting questionnaire that is a quantitative measure of distress. The presence of symptoms over the previous week was modified to 2 weeks prior to the endoscopy for the purposes of this study for logistical reasons related to problems with bookings. The scale assesses negative emotional symptoms by using a 4-point Likert scale (never applies to me = 0, to almost always applies to me = 3) and has three subscales consisting of seven items each for depression (D), anxiety (A) and stress (S). As the DASS-21 is a shortened version of the original tool that had 42 items, the final score of each item group (D, A, S) is multiplied by two. Screen negative scores for D, A and S are < 10, < 8 and < 15, respectively.^29^

**FIGURE 1:**
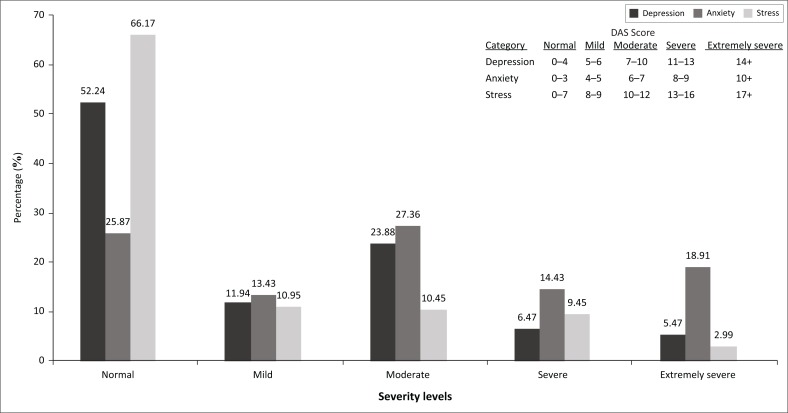
Frequency of symptoms and severity for depression, anxiety and stress.

The cut-off scores based on five categories (normal, mild, moderate, severe and extremely severe) for DAS are noted in Figure 1. Although the emotional symptoms being quantified are dimensional, categorical severity labels are available.^30,31^ The DAS scores correlate with DSM or ICD diagnostic categories, and indicate the degree of difference between the A and the S experienced by normal subjects and clinical populations.^29^ The DASS-21 has been validated across different countries in both clinical and community populations, and has been shown to have a good correlation with the Beck Depression and Anxiety scale.^32,33^ Study participants who screened as being severely or extremely anxious or depressed received a referral letter for further treatment.

### Statistical analyses

Following a descriptive analysis of the participants’ socio-demographic and clinical details (i.e. to describe the frequency of DAS), linear regression models were fitted to identify potential risk and protective correlates linked to DAS symptomatology. A *p*-value of less than 0.05 was considered to be statistically significant, with the data being entered into SPSS version 24 and the analyses conducted using STATA 15.

### Ethical considerations

Approval was obtained from University of KwaZulu-Natal Biomedical Research Ethics Committee BREC reference number: BE374/16.

## Results

### Socio-demographic and clinical profiles

Of the 209 participants who were identified, eight were excluded due to incomplete data, which resulted in the analysis being conducted on 201 respondents. The socio-demographic and clinical profiles of participants are presented in Tables 1 and 2. Their mean age was 48.89 years, two-thirds were female and 97% were African. The majority (64.68%) resided in rural areas and 80.5% received a monthly income of < R3000. Almost a quarter reported alcohol (*n* = 42; 21%) or substance use that included cigarettes and cannabis (*n* = 46; 22.89%). More than half had a previous history of dyspepsia (*N* = 105), while endoscopic abnormalities were detected in 167 (89.78%) and hiatus hernia in 11. The commonest finding was gastritis (*N* = 86) alone or with other conditions (oesophagitis, duodenitis, gastric or duodenal ulcers). Peptic or duodenal ulcers were present in 19 and tumours in 15 participants, with the histopathological results not being available at the time of the study.

**TABLE 1:** Socio-demographic profiles.

Variable	*N*	%
**Gender**		
Male	68	33.83
Female	133	66.17
**Age**		
Under 30	39	18.75
30–59	101	48.56
60+	68	32.69
**Race and/or ethnicity**		
African	195	97.01
Mixed race	3	1.49
Indian	2	1.00
White	1	0.50
**Education**		
None or primary	94	47.24
High school or greater	105	52.76
**Marital status**		
Single	96	47.76
Married	63	31.34
Divorced or widowed or other	42	20.9
**Household income**		
< R3000	161	80.5
≥ R3000	39	19.5
**Living alone**		
Yes	11	5.5
No	189	94.5
**No. of household members**		
0–4	83	41.29
5–9	102	50.75
10+	16	7.96
**Residential setting**		
Urban	64	31.84
Rural	130	64.68
Informal	7	3.48

**TABLE 2:** Clinical profiles.

Variable	*n*	%
**HIV**		
Yes	49	24.38
No	86	42.79
Unknown status	66	32.84
**Alcohol abuse**		
Yes	42	21.00
No	158	79.00
**Substance abuse**		
Yes	46	22.89
No	155	77.11
**Chronic medical illness (excluding mental illness)**		
Yes	137	68.84
No	62	31.16
**Endoscopy results**		
Normal	19	10.22
Abnormal	167	89.78
**Previous dyspepsia**		
Yes	105	52.24
No	96	47.76

**FIGURE 2:**
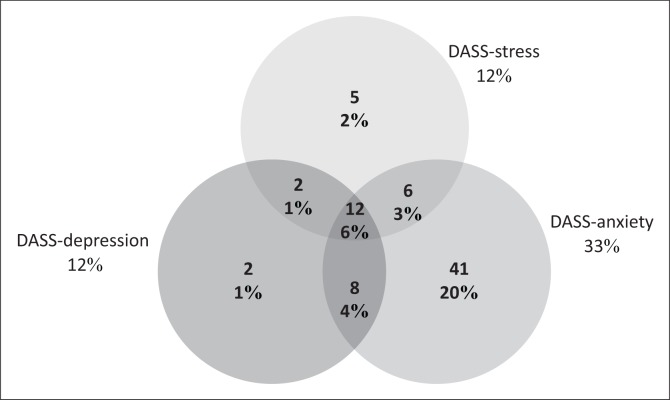
Severe and extremely severe D (depression), A (anxiety) and S (stress) comorbidities.

Regarding the frequency of DAS, ranging from mild to extremely severe, they were present in 96 (47.76%), 149 (74.13%) and 68 (33.83%) participants, respectively. Anxiety was the most prevalent of the three symptom categories overall as well as in each category of severity. The total numbers of participants scoring in the combined severe and extremely severe ranges were 24 (11.94%), 67 (33.33%) and 25 (12.44%) for DAS, respectively (Figure 1). Figure 2 represents the frequency of the comorbidities of DAS in the combined severe and extremely severe ranges, and while depression (2/24; 8.33%) and stress (5/25; 20%) rarely occurred in isolation, anxiety was present as a single symptom category in 41/67 (61.19%) of the severe and extremely severe anxious patients.

Using logistic regression analyses (Table 3), the following negative correlates of DAS were detected: males with dyspepsia were significantly more anxious than females.

**TABLE 3:** Regression models investigating risk and protective correlates of depression, anxiety and stress.

Variables	Depression			Anxiety			Stress			Total domains (0–3)		
	Adj *β*	SE	*p*	Adj *β*	SE	*p*	Adj *β*	SE	*p*	Adj *β*	SE	*p*
**Gender (Female)**												
Male	0.95	1.59	0.55	3.98	1.39	<0.01	0.44	1.69	0.79	0.23	0.16	0.15
**Age category (< 30)**												
30 – 59	0.46	1.99	0.82	−1.52	1.74	0.39	−1.24	2.11	0.56	−0.37	0.20	0.06
60+	−1.56	2.71	0.56	−3.18	2.38	0.18	−3.22	2.89	0.27	−0.50	0.27	0.07
**Education (None or primary)**												
High school or greater	3.59	1.72	0.04	1.39	1.51	0.36	4.80	1.83	0.01	0.30	0.17	0.08
**Marital status (Single)**												
Married	1.66	1.69	0.33	3.22	1.48	0.03	3.55	1.80	0.05	0.42	0.17	0.01
Divorced/widowed/other	1.83	1.99	0.36	2.39	1.75	0.18	1.95	2.12	0.36	0.30	0.20	0.13
**Income category (< R3000/month)**												
≥ R3000/month	−1.88	1.77	0.29	−3.45	1.56	0.03	−4.30	1.89	0.02	−0.17	0.18	0.35
**Living alone (Yes)**												
No	5.00	2.86	0.08	4.28	2.51	0.09	4.24	3.05	0.17	0.37	0.28	0.19
**Family composition (0 – 4)**												
5 – 9	0.50	1.34	0.71	0.17	1.18	0.88	1.15	1.42	0.42	0.05	0.13	0.70
Greater than 10	−0.46	2.48	0.85	−3.28	2.18	0.13	−1.80	2.64	0.50	−0.25	0.25	0.31
**Typology (Urban)**												
Rural	−1.67	1.38	0.23	−0.20	1.21	0.87	−0.39	1.47	0.79	−0.03	0.14	0.83
Informal	1.89	3.82	0.62	−2.05	3.35	0.54	4.99	4.06	0.22	−0.33	0.38	0.38
**HIV status (Yes)**												
No	−0.28	1.97	0.89	0.29	1.73	0.87	−1.36	2.10	0.52	−0.04	0.20	0.84
Unknown	−3.01	2.92	0.30	−1.00	2.56	0.70	−3.43	3.10	0.27	−0.38	0.29	0.19
**Alcohol risk (Yes)**												
No	−7.54	2.27	<0.01	−7.49	1.99	<0.01	−6.54	2.42	<0.01	−0.90	0.22	<0.01
**Substance use risk (Yes)**												
No	2.09	2.21	0.35	1.53	1.94	0.43	1.04	2.35	0.66	0.14	0.22	0.51
**Chronic medical condition (Yes)**												
No	2.12	2.56	0.41	−1.19	2.25	0.60	1.15	2.72	0.67	0.15	0.25	0.54
**Endo result (Normal)**												
Abnormal	0.07	2.14	0.97	−0.02	1.88	0.99	−0.09	2.28	0.97	0.07	0.21	0.76
**Previous dyspepsia (Yes)**												
No	−2.94	1.29	0.02	−2.14	1.14	0.06	−1.23	1.38	0.37	−0.25	0.13	0.05

Note: Reference category in bracket.

Higher education level (> primary level) was significantly associated with depression and stress. Alcohol abuse was significantly associated with DAS. In terms of positive correlates, our logistic regression analyses detected the following: higher income and being married was significantly associated with anxiety and stress. No history of previous dyspepsia was also correlated with lower depressive symptoms. Age, living arrangement, family composition, typology, HIV status, substance use, chronic medical conditions and endoscopic abnormalities were not significantly associated with depression, anxiety or stress.

## Discussion

Dyspepsia and CMD symptoms are frequent clinical presentations that are known to be co-morbid. The aim of this study was therefore to describe the frequency of DAS and their associated preventive and risk correlates in patients undergoing endoscopy for dyspepsia at a regional public sector hospital in KZN. The frequency of symptoms in excess of normal was reported by between one-third (stress) and three-quarters (anxiety) of the study sample.

The study reported on a clinical sample characterised by mainly older (mean 48.89 years) female participants from predominantly rural backgrounds and with low monthly income. Being a male was associated with an increased risk of anxiety (*p* = 0.02), and although female gender is a proposed risk correlate, a meta-analysis of risk factors found it to be only modestly associated with uninvestigated dyspepsia, but not in African studies.^34^ All anxiety disorders were more prevalent among females across the lifespan with genetic, neurodevelopmental, environmental and neurobiological factors implicated in the gender differences.^35^

Among the risk factors associated with CMDs of primary care patients in developing countries are older age, female, being widowed, separated or divorced, unemployment, low socioeconomic status, alcohol problems and chronic medical illness, including peptic ulcer.^1,36,37^ Anxiety in non-clinical community samples in South Africa is also more common in females,^38^ with studies in a larger sample being required to confirm and understand the reasons for dyspeptic males’ heightened anxiety levels.

Alcohol was the most commonly used substance in our sample and was significantly associated with DAS (*p* < 0.01 for each). A review of three studies concluded that a high intake of alcohol may lead to the development and aggravation of functional GI disorders, especially FD, this being a dose-dependent relationship.^39^ In a Nigerian study, alcohol was found to be a risk factor for FD,^40^ with almost 50% of males consuming alcohol, which accounts for 7.0% of Disability Adjusted Life Years in South Africa. There is a strong sociopolitical background to alcohol use in South Africa,^41^ and given the association with anxiety, dyspepsia and its use among males in our study, exploring the role of alcohol in the onset and course of dyspepsia locally could offer insights into the latter’s management and prognosis.

Our findings of above normal, all-severity levels of DA and S in 47.76%, 74.13% and 33.83%, respectively, are lower than that reported by Haider et al.^18^ using the DASS in Pakistan. While the DASS does not correlate with formal psychiatric diagnoses, the high frequency of anxiety, in particular, in our study is worthy of further clinical attention in dyspepsia patients; in a community survey of mental disorders in South Africa, anxiety was the most prevalent class of lifetime disorders (15.8%) across all age groups, with mood disorders at 9.8%.^38^ High prevalence rates of CMDs have been reported in developing countries,^1,36^ with a prevalence of generalised anxiety and depressive disorders of 23.9% in a community-based sample in South Africa.^36^

The high frequency of anxiety in dyspepsia patients has implications for whether sufferers choose to seek medical attention. In one study, consulters were distinguished by concern (worry) about the possible seriousness of the symptoms rather than the severity of their abdominal symptoms. Greater adverse events and over-representation by lower social class were characteristics of patients who consulted doctors for dyspepsia.^42^ Similarly, anxiety, but not depression, was found to be a significant independent factor in determining consultation in patients with dyspepsia in a Chinese population survey.^43^ In a small sample, FD patients were shown to have significantly higher scores than healthy controls in psychosocial stress, mood (stress, anxiety, depression), coping styles, type A personality and half-time of gastric emptying-findings that implicate stress in the a etiopathogenesis of dyspepsia.^44^

Functional dyspepsia patients were reported to have different regional brain activities on functional brain imaging, with abnormalities in central visceral pain processing implicated in FGID.^45^

Recognition of the need for a holistic approach to the aetiology and assessment of dyspepsia is to be found in the change of FGIDs to ‘disorders of gut–brain interaction’, with the Rome IV diagnostic criteria having a multicultural rather than a Western-culture focus. Guideline chapter on the ‘Psychosocial Aspects of Functional Gastrointestinal Disorders’ has been changed to the ‘Biopsychosocial Aspects of Functional Gastrointestinal Disorders’.^46^ The implications of psychosocial factors for treating dyspepsia are also being recognised, with lifestyle changes, antidepressants and psychological therapies appearing in treatment recommendations and guidelines, although there is limited evidence to date on the effect of such management strategies.^20,47,48,49^

Apart from its clinical implications, the additional impact of the co-morbidity of dyspepsia and psychiatric symptoms on work absenteeism and costs (direct and indirect) has relevance. Employees with FD have been shown to have greater health-related costs, absenteeism and lower productivity than those without.^28^ The possible contribution of co-morbid CMD to absenteeism has implications for the use of psychotropic medication to reduce the burden of dyspepsia for individuals and society.

### Limitations of the study

Our sample size was small and confined to patients awaiting endoscopy at a regional hospital.

No sample size was calculated for this study and our results are not generalisable to community samples.

Furthermore, not all race groups were represented, with the findings therefore not being reflective of the general South African population. The DASS-21 is a screening tool, and although the presence of DAS symptoms cannot be equated to clinical diagnoses, the findings support the need for further studies using diagnostic tools to establish the association of major or minor depression and GAD in patients with dyspepsia. Finally, although the patients met the Rome III diagnostic criteria for functional dyspepsia, they were not classified into subtypes, each of which may have different associations with psychiatric symptoms.

## Conclusion

Proper screening for CMDs is essential in individuals with dyspepsia and vice versa. The role of psychiatric factors in the aetiology, course and management of dyspepsia requires further exploration, particularly in the local context and lower socio-economic settings, to provide holistic clinical care and optimise outcomes.
